# Machine Learning Approaches to Classify Primary and Metastatic Cancers Using Tissue of Origin-Based DNA Methylation Profiles

**DOI:** 10.3390/cancers13153768

**Published:** 2021-07-27

**Authors:** Vijayachitra Modhukur, Shakshi Sharma, Mainak Mondal, Ankita Lawarde, Keiu Kask, Rajesh Sharma, Andres Salumets

**Affiliations:** 1 Competence Centre on Health Technologies, 50411 Tartu, Estonia; modhukur@ut.ee (V.M.); matrix.mainak994@gmail.com (M.M.); ankita.sunil.lawarde@ut.ee (A.L.); keiu.kask@ut.ee (K.K.); 2 Department of Obstetrics and Gynecology, Institute of Clinical Medicine, University of Tartu, 50406 Tartu, Estonia; 3 Institute of Computer Science, University of Tartu, 51009 Tartu, Estonia; shakshi.sharma@ut.ee (S.S.); rajesh.sharma@ut.ee (R.S.); 4 Division of Obstetrics and Gynecology, Department of Clinical Science, Intervention and Technology (CLINTEC), 14186 Stockholm, Sweden

**Keywords:** DNA methylation, TCGA, biomarkers, clustering, differential methylation, metastasis, epigenetics, machine learning, artificial intelligence, explainable predictions

## Abstract

**Simple Summary:**

Cancer metastasis is considered to be one of the most significant causes of cancer morbidity, accounting for up to 90% of cancer deaths. The accurate identification of a cancer’s origin and the types of cancer cells it comprises is crucial in enabling clinicians to decide better treatment options for patients. DNA methylation changes are increasingly recognized as determining cancer prediction, especially for the transition to metastasis. Research in the last decade has shown the incredible promise of the use of artificial intelligence (AI) in cancer classification. In this study, we applied several machine learning techniques, a branch of AI, to identify cancer tissue or origin and further classified cancer samples as primary and metastatic cancers based on publicly available DNA methylation data. Overall, our analysis resulted in a 99% accuracy for predicting cancer subtypes based on the tissue of origin.

**Abstract:**

Metastatic cancers account for up to 90% of cancer-related deaths. The clear differentiation of metastatic cancers from primary cancers is crucial for cancer type identification and developing targeted treatment for each cancer type. DNA methylation patterns are suggested to be an intriguing target for cancer prediction and are also considered to be an important mediator for the transition to metastatic cancer. In the present study, we used 24 cancer types and 9303 methylome samples downloaded from publicly available data repositories, including The Cancer Genome Atlas (TCGA) and the Gene Expression Omnibus (GEO). We constructed machine learning classifiers to discriminate metastatic, primary, and non-cancerous methylome samples. We applied support vector machines (SVM), Naive Bayes (NB), extreme gradient boosting (XGBoost), and random forest (RF) machine learning models to classify the cancer types based on their tissue of origin. RF outperformed the other classifiers, with an average accuracy of 99%. Moreover, we applied local interpretable model-agnostic explanations (LIME) to explain important methylation biomarkers to classify cancer types.

## 1. Introduction

The term “metastasis” can be defined as the spread of cancer cells to surrounding tissues and distant organs from the primary site the cancer originated from. Metastasis is suggested to be the major cause of cancer morbidity and mortality, accounting for about 90% of cancer deaths [1]. Due to this, the stage and type of metastasis determine the overall cancer prognosis [2]. For instance, distant metastases are suggested to mark stage IV of carcinogenesis, resulting in a relatively shorter overall survival of the patient [3]. Advances in early cancer detection and subsequent therapies have enabled us to improve tumor management; however, once cancers spread beyond the initial primary site, personalized cancer treatment becomes very challenging. Therefore, it is crucial to accurately identify different cancer types as well as their primary origin.

In cancer prediction, immunohistochemistry (IHC) is usually carried out to determine the cancer origin or to bring out the variation between different types of cancer. This method uses enzyme or fluorescent dye-linked antibodies for certain antigens (target proteins) in a tissue sample. When the antibody binds to a target protein, the enzyme or dye is activated, and the existence and localization of the target protein can be seen under a microscope [4]. Although it is widely used, IHC has a rather limited accuracy (66–88%) in determining the origins of metastatic cancer, and its use is limited to known proteins [5]. There is no single antibody that is absolutely site-sensitive and site-specific for the prediction of the primary site of cancer; therefore, a cocktail of multiple biomarkers is needed for reaching a correct diagnosis [6]. Usually, if the morphological analysis is not sufficient, the diagnostic IHC panel approach is used, which includes antibodies against epithelial antigens (AE1/AE3, KL1), lymphoid antigens (CD45, CD20, CD3), and melanocyte-differentiation antigens (S100 protein, SOX10) [7]. In addition, a myriad of useful biomarkers has been suggested to be useful in the IHC panels, such as cytokeratin 7 (CK7), CK 20, estrogen receptor (ER), mammaglobin (MGB), thyroid transcription factor 1 (TTF1), caudal type homeobox 2 (CDX2), Wilms tumor 1 (WT1), napsin A, gross cystic disease fluid protein 15 (GCDFP-15), octamer-binding transcription factor 4 (OCT4), paired box gene 8 (PAX8), GATA binding protein 3 (GATA3), arginase 1 (ARG1), and trefoil 1 (TFF1) [8]. Thus, the selection of an appropriate IHC panel, a wide variety of protocols, and different methods for preanalytical acquisition and handling of the sample could affect the overall outcome in addition to the subjective interpretation of the results [8].

DNA methylation is an epigenetic modification whereby a methyl residue is added to the cytosine nucleotides, which is an essential hallmark for cancer initiation [9]. In particular, certain tumor suppressor genes are inactivated due to hypermethylation within the promoter region of the gene [10]. Contrarily, global hypomethylation induces genomic instability and contributes to cancer cell transformation [10,11]. DNA methylation changes in cancer have been illustrated in a few longitudinal studies [12,13,14]. DNA methylation changes are also common in the transition from one cancer stage to another [15]. Furthermore, DNA methylation changes are suggested to be an important mediator for the transition to metastatic cancers and have also been highlighted in prospective cohort studies [16].

DNA methylation patterns are established in a tissue-specific manner in both normal tissues [17] and tumor tissues [18]. In this direction, multiple studies have focused on identifying tumor types based on DNA methylation patterns. For instance, Moran et al. developed a diagnostic tool for the identification of tumor of origin, EPICUP [19], which compares the methylation profiles of CUP (cancer of unknown primary origin) with those of known primary tumors and their matched metastasis methylation profiles to identify the tumor origin [20]. The overall accuracy of EPICUP was reported to be 87% [19]. On the other hand, CancerLocator predicts cancer types and tissue origins using the cell-free DNA (cfDNA) methylation profiles from blood samples [21]. Briefly, CancerLocator learns informative features from TCGA, followed by modeling normal cfDNA and circulating tumor DNA (ctDNA). Furthermore, CancerLocator achieved a Pearson’s correlation coefficient (PCC) of 0.975 between the predicted and actual proportions of ctDNA [21]. Another study by Daniel Xia et al. [22] introduced a minimalistic approach for DNA methylation-based cancer diagnosis. The minimalistic approach combines both TCGA and internal methylation profiling data and uses only 53 CpGs for tumor classification, with an overall accuracy of 94.5% across 28 cancer types when using TCGA data [22]. Similarly, Chen et al. used a clustering approach for the prediction of urological cancers using DNA methylation, copy number, and RNA and protein expression [23]. In another study, Tang et al. [24] used a combination of miRNA and DNA methylation to predict cancer origin using a machine learning approach. In a recent study, Zheng et al. [5] used a deep neural network to predict cancer origin based on DNA methylation patterns, with an overall accuracy of 99.72%. Several of the above-mentioned classifiers used a wide range of AI-based approaches, such as deep learning and machine learning methods. However, they lacked interpretability due to their “black-box” nature. The current study aims to classify different methylome samples on the tissue of origin and predict whether the given methylome sample represent the tumor or normal tissue. Furthermore, if the given sample is a tumor, we predict whether the tumor type is primary or metastatic based on CpG methylation levels using a multi-label machine learning classifier. In addition, our study also aims to provide explanations for the prediction features by applying local interpretable model-agnostic explanations (LIME).

## 2. Materials and Methods

### 2.1. DNA Methylation Data Collection

**Figure 1:**
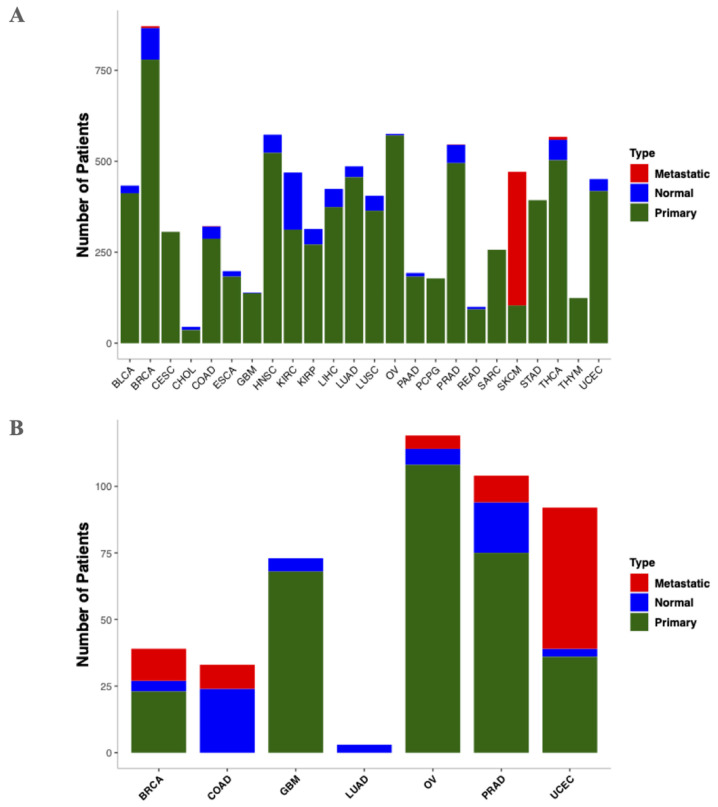
Bar plot for depicting the number of methylome samples used in this study. (**A**) Samples from TCGA. (**B**) Samples from GEO. Blue color bars indicate normal samples, while green and red denote primary and metastatic tumors, respectively. BLCA: bladder urothelial carcinoma; BRCA: breast invasive carcinoma; CESC: cervical squamous cell carcinoma and endocervical adenocarcinoma; CHOL: cholangiocarcinoma; COAD: colon adenocarcinoma; ESCA: esophageal carcinoma; GBM: glioblastoma multiforme; HNSC: head and neck squamous cell carcinoma; KIRC: kidney renal clear cell carcinoma; KIRP: kidney renal papillary cell carcinoma; LIHC: liver hepatocellular carcinoma; LUAD: lung adenocarcinoma; LUSC: lung squamous cell carcinoma; OV: ovarian cancer; PAAD: pancreatic adenocarcinoma; PCPG: pheochromocytoma and paraganglioma; PRAD: prostate adenocarcinoma; READ: rectum adenocarcinoma; SARC: sarcoma; SKCM: skin cutaneous melanoma; STAD: stomach adenocarcinoma; THCA: thyroid carcinoma; THYM: thymoma; UCEC: uterine corpus endometrial carcinoma.

Methylome data consisting of primary tumors, metastatic tumors, and the matched normal samples, including 24 cancer types, were collected from TCGA and the GEO. The UCSC Xena browser [25] was used to download methylation data based on TCGA, while the R package GEOquery [26] was used to download the methylation data from GEO. In this study, methylation data from both the Illumina Infinium HumanMethylation27 (27K) and the Illumina HumanMethylation450 BeadChip (HM450K) methylation platforms were used. As suggested by Zheng et al. [5], compatible features from 27K were added to reduce the dimensionality presented in the HM450K array. In total, 9303 (TCGA = 8840 and GEO = 463) patient samples were used for this study. Figure 1A,B denotes the total number of samples used in this study from (A) TCGA and (B) GEO. The samples were grouped into three types: normal tissues and primary and metastatic tumors. Detailed phenotype information for the methylome samples used in the study and their corresponding accession codes are presented in Table S1.

### 2.2. Preprocessing and Processing of Methylation Samples

The DNA methylation level of the CpG site was expressed as a beta value. Methylation beta values ranged between 0 and 1, with 0 being unmethylated and 1 being fully methylated. The beta values were derived from methylated and unmethylated probe intensities using the formula M/(M + U + 100), where M and U are fully methylated and fully unmethylated intensities, respectively [27]. The methylome data were further normalized using the function “normalizeWithinArrays” from the Bioconductor package (version number 3.13) package limma (version number 3.48.0) [28]. The missing values were imputed using the K-nearest neighbor (KNN) approach for each cohort [29]. Probes with >50% missing values (N = 3331) were removed. We further filtered out probes mapped to sex-specific chromosomes (N = 1092) and cross-reactive probes (N = 2597). A total of 20,558 CpGs were used for subsequent analysis.

The CpG sites were annotated to genomic regions according to the annotation file (genome build 37) provided by Illumina.

### 2.3. Identification of Methylation-Based Cancer Biomarkers

A linear model fit was performed using the Bioconductor (version number 3.13) package limma (version number 3.48.0) [28] to identify differentially methylated CpG sites (DMS). The DMS analyses were performed to assess the differences in methylation levels between (i) normal and primary and (ii) primary and metastatic methylome samples. The linear model fit by limma used moderated t-statistics, where the standard errors were moderated using an empirical Bayes model. The Benjamini–Hochberg FDR was calculated for each CpG, with an FDR-corrected *p*-value < 0.05 used to define a DMS. Further, to obtain optimal DMSs, our results were limited to the absolute mean methylation differences between the compared groups (delta beta) >0.2.

### 2.4. Machine Learning Models to Classify Cancers

In the current study, four machine learning classifiers were used for cancer classification: SVM (support vector machine), Naive Bayes (NB), random forest (RF), and extreme gradient boosting (XGBoost). Among the methods mentioned above, random forest (RF) and extreme gradient boosting decision trees (XGBoost) are the most popular ensemble learning methods [30]. Moreover, both RF and XGBoost account for correlation and interaction among features. On the other hand, SVM was used to find a hyperplane in a feature space that can separate or classify the samples. A loss function is defined to maximize the margin; that is, to find the maximum distance between multiple classes [30]. Conversely, NB is a Bayesian approach of supervised classification that assumes that the probability of one feature predicting a class is independent of other features [31].

SVM models were implemented using the python package “sklearn.svm.SVC” (version number 0.24.2) using the kernel “rbf” and the parameters “C-1”, “degree—3” and the decision function shape “ovr”. NB was implemented using the “GaussianNaiveBayes” package (version number 0.24.2). XGBoost was implemented using the package “xgboost” (version number 1.4.0), with the number of trees set to 100. RF was implemented using the package “sklearn.RandomForestClassifier” (version number 0.24.2) with the number of trees set to 100. The criterion “gini” and the minimum number of samples split was set to two, and the minimum samples leaf was set to one.

### 2.5. Dimensionality Reduction Using t-SNE (T-Distributed Stochastic Neighbor Embedding) and Principal Component Analysis (PCA)

The t-SNE technique was used to visualize methylome data sets that displayed high-dimensional data, providing each data point with a location in two-dimensional (2D) space. In 2D, similar objects tend to cluster together, while dissimilar ones are modeled to distant points. In addition to t-SNE, PCA was used to visualize high-dimensional methylome data. In principle, PCA reduces the high dimensionality by identifying the directions known as principal components along which the data variation is maximized [32].

### 2.6. Splitting of the Data Sets

The 9303 samples used in this study were randomly split into an 80:20 ratio. In other words, 80% of the data was used for training the model, while 20% was used for testing the model that was built from it. To avoid potential bias, cross-validation was performed in five random folds (K-Fold cross-validation).

### 2.7. Balancing the Methylome Samples Using SMOTE

Since the number of methylome samples from each class and type of cancer was not balanced, the synthetic minority oversampling technique (SMOTE) [33] was used with the python package “scikit-learn” (version number 0.24) to obtain balanced data.

### 2.8. Evaluation Metrics

The performance of each of the classification models was evaluated according to its accuracy, sensitivity, specificity, precision, recall, and F1-score. Accuracy describes the correct classifications out of all classifications in the range of 0–100%. Sensitivity is a measure that describes the proportion of positives that are correctly identified. Sensitivity is also known as the true positive rate, and the values range from 0% to 100%. Specificity, also known as the true negative rate, describes the proportion of correctly identified negatives. Sensitivity values also range from 0% to 100%. Precision is a measure that describes how many cases are actual cases among all the cases predicted. The precision value ranges from 0 to 1. Recall is a measure that specifies how many cases we were able to identify as actual cases. The recall value ranges from 0 to 1. F1-score is the weighted average of precision and recall. The F1-score ranges from 0 to 1.

In addition to the above-mentioned performance metrics, the classifier results were evaluated using receiver operator curve (ROC) representation. ROC describes the sensitivity and specificity of a test. In a typical ROC, the x-axis depicts the negative predictions while the y-axis depicts the positive predictions [34].

### 2.9. Local Interpretable Model-Agnostic Explanations (LIME) for Machine Learning Model

LIME was used to understand the classifier’s decision. LIME is an AI explainability tool used by machine learning models to understand the reasoning behind a model’s decision. Since several machine learning classifiers use representations that are black-box in nature, LIME explains the reasoning behind the classifier’s decision by perturbing the input samples around the neighborhood and see the model’s prediction behavior [35]. The python package “lime” was used to implement the LIME-related functions in this study.

### 2.10. Gene Ontology, Pathway Analysis, and Network Visualizations

Gene Ontology (GO) and Kyoto Encyclopedia of Genes and Genomes (KEGG) pathway enrichment analyses of differentially methylated sites (DMS) were performed using the webtool Metascape [36]. In addition, a protein–protein (PPI) interaction analysis was performed using Metascape. The FDR-corrected *p*-value < 0.05 was considered to be statistically significant. The Human Cancer Metastasis Database (HCMDB) [37] and Human Protein Atlas (HPA) database [38] were also used to evaluate the methylation biomarkers used as features for the classification of cancer types.

## 3. Results

### 3.1. DNA Methylation Biomarkers for Cancer Type Classification

**Figure 2:**
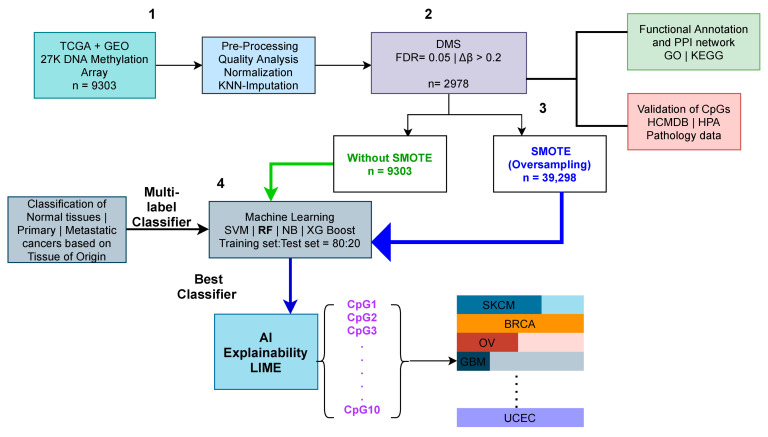
Schematic representation of analysis presented in the study. The overall workflow is divided into four components: (**1**) data acquisition, preprocessing, and quality control analysis; (**2**) defining prediction biomarkers for the classification using DMS analysis; (**3**) evaluation of predictive biomarkers using GO, KEGG, and PPI analysis; and (**4**) applying machine learning models and interpreting the decision of the classifier using LIME. A total of 9303 methylome samples were download from TCGA and GEO. The samples were preprocessed and normalized, and missing values were imputed using the KNN method. Further, DMS analysis was performed to define prediction biomarkers. The biomarkers were evaluated using GO, KEGG, and PPI analysis and further compared with the HCMBD and HPA pathology databases. A multi-label ML classification model was built for classifying normal tissues, and primary and metastatic cancer types based on tissue of origin, before and after applying SMOTE. LIME was applied to the best-performing model to interpret the prediction model for every class.

An overview of the workflow used in this study is shown in Figure 2. The workflow consisted of four main components: (i) data acquisition from TCGA and GEO, and preprocessing and quality control analysis; (ii) defining biomarkers for the classification using DMS analysis; (iii) the evaluation of predictive biomarkers using GO, KEGG, and PPI analysis through Metascape [36] and HPA [38]; and (iv) applying machine learning models to classify normal tissue, and primary and metastatic cancer types based on tissue of origin, before and after applying SMOTE, using a multi-label classifier, and interpreting the decision of the classifier using LIME. The results from each of the components mentioned above are described in detail in the following sections.

After the data processing was complete, differential methylation analysis was performed to select the most informative CpGs as methylation-based biomarkers for cancer type classification. Our analysis resulted in 2978 CpGs (Table S2) using the cut-off of the FDR-corrected *p*-value < 0.05 and the absolute mean methylation difference between the compared groups (delta beta) of >0.2.

Next, we used the t-SNE technique to visualize the methylation biomarkers used for classifying cancer methylome data sets that display high-dimensional data in a 2D space. According to the t-SNE visualization (Figure S1), we observed that cancer samples cluster according to their tissue type. For instance, kidney-related methylome samples, ovarian methylome samples, and liver samples were clustered together (Figure S1). Likewise, uterine samples were clustered together. Moreover, samples from breast tissue formed a unique cluster with distinguishable normal tissue and primary and metastatic cancer sample types (Figure S1). In addition to t-SNE, we used PCA to visualize the clustering patterns of DNA methylation biomarkers. However, PCA (Figure S2) did not cluster the samples well enough in comparison to t-SNE. Overall, t-SNE showed generally well-separated clusters according to our analysis.

### 3.2. Construction of Machine Learning Models

In the current study, altogether, we used 9303 methylome samples processed from the TCGA and GEO methylome data sets. Our data sets included 24 different cancer types, including normal, primary, and metastatic labels based on the tissue of origin. We attempted to build a multi-label machine learning classifier using 2978 methylation biomarkers to classify cancer types consisting of 49 class labels.

We applied four machine learning models: SVM, Naïve Bayes (NB), extreme gradient boosting (XGBoost), and random forest (RF). Using five-fold cross-validation (Table S3), the XGBoost method resulted in the highest testing and training accuracy (90%). By contrast, the NB method resulted in the lowest accuracy (78%). However, both the SVM and RF methods resulted in a >88% accuracy (Table S3).

### 3.3. Classifier Performance after Balancing the Methylome Data Sets Using SMOTE

As we discovered, the number of samples per class was highly imbalanced; therefore, we applied the SMOTE technique to balance the data set. After SMOTE, the total number of methylomes was 39,298. We further tested the classifier’s performance after performing SMOTE and noted that the overall accuracy, precision, recall, and F1-score had increased by almost 10%. Moreover, the RF classifier showed the highest performance, with >0.98 for accuracy, precision, recall, and ROC/AUC score among all the classifiers tested (Table S3). We found a precision and recall is higher than 0.97 (Table 1) in the majority of cancer types, except for lung squamous cell carcinoma—primary; head and neck squamous cell carcinoma—primary; and stomach adenocarcinoma—primary. Interestingly, for many cancer types, including bladder, brain, colorectal, esophageal, kidney, ovary, thyroid, and stomach, the RF model achieved precision and recall of 100% (Table 1). The confusion matrix and ROC/AUC curve are shown in Table S4 and Figure S3, respectively. Overall, the average accuracy of the RF classifier was 99% (Table 1).

### 3.4. Interpreting Model Predictions through LIME

**Figure 3:**
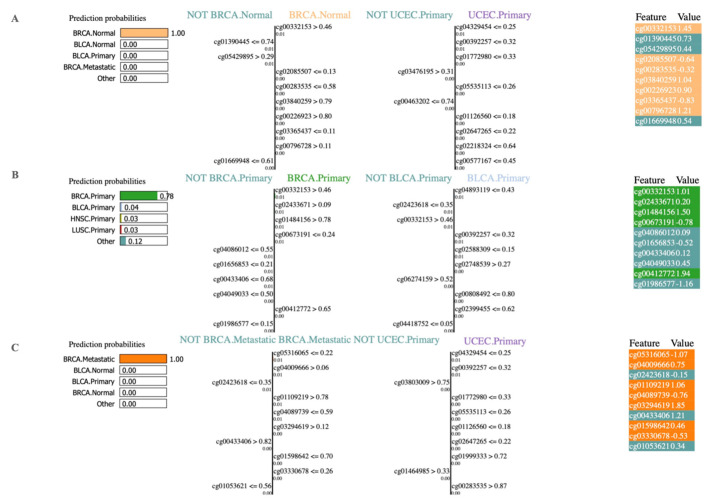
Results of LIME for top 10 biomarkers using RF classifier illustrated for normal breast, primary breast cancer, and metastatic breast cancer. (**A**) Lime interpretations for normal breast. The blue color denotes the negative instance, and the orange color denotes the positive instance. The first column represents the prediction probabilities of negative and positive results achieved from classifiers. The second column shows the features’ contributions to the probability. The third column displays the original data values. (**B**) LIME interpretation for primary breast cancer. (**C**) LIME interpretation for metastatic breast cancer. LIME: local interpretable model-agnostic explanations; RF: random forest.

We applied LIME to obtain interpretable machine learning predictions and to further investigate the feature contributions. In the current study, 2978 methylation biomarkers were used as features for the prediction of cancer types. The LIME predictions of the RF model for the classification of normal, primary, and metastatic breast cancer samples using the top 10 prediction biomarkers are illustrated in Figure 3. The top-left corner indicates the prediction probability for predicting normal breast tissue, shown as BRCA.Normal, ranging from 0 to 1. In addition, the feature combination for not normal breast, shown as Not BRCA.Normal|BRCA.Normal and not primary uterine cancer, shown as Not UCEC.primary|UCEC.primary are shown as separate tables. Here, LIME assigned 0.01 as the feature weight for Cg0332153 > 0.46. Likewise, feature weights are shown for the other top predicting features. Finally, in the top-right corner, each feature is color-coded to specify whether a given feature contributes to the prediction. The normal breast tissue prediction (BRCA.Normal) is shown in orange, while not normal breast tissue (Not BRCA.Normal) is color-coded in green (Figure 3).

Similarly, we illustrate the interpretation of LIME for primary (Figure 3B) and metastatic (Figure 3C) breast cancer types. The prediction probability for predicting primary and metastatic breast cancer ranges from 0.03 to 0.78 and 0 to 1, respectively. The feature weights are also shown further. The prediction for primary breast cancer is shown in green color, while the negative prediction is shown in blue color. Likewise, the prediction for metastatic breast cancer is shown in orange color, while the negative prediction is shown in blue color. The LIME interpretations for other metastatic cancer types used in this study are shown in Figure S4.

### 3.5. Gene Set Enrichment Analysis and PPI Enrichment of Methylation Biomarkers Additionally, Its Relation to Metastasis

**Figure 4:**
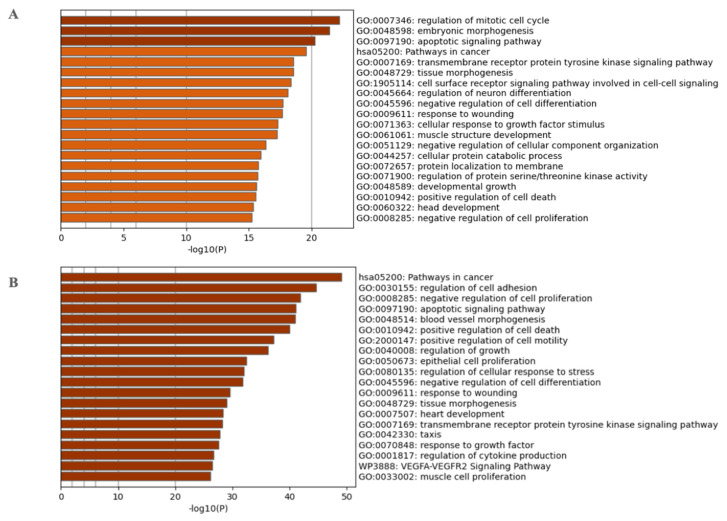
Top GO and KEGG terms using Metascape for (**A**) 2737 genes annotated to methylation biomarkers and (**B**) for 383 metastatic genes mapped using HCMDB.

The 2978 CpGs used as prediction biomarkers were annotated to 2737 genes. The top 20 enriched GO and KEGG pathway terms according to Metascape are shown in Figure 4A,B, respectively, and Table S5. The top 20 enriched GO and KEGG pathway terms include the regulation of the mitotic cell cycle, embryonic morphogenesis, the apoptotic signaling pathway, pathways in cancer, the transmembrane receptor protein tyrosine kinase signaling pathway, tissue morphogenesis, the cell surface receptor signaling pathway involved in cell–cell signaling, the regulation of neuron differentiation, the negative regulation of cell differentiation, response to wounding, cellular response to growth factor stimulus, muscle structure development, the negative regulation of cellular component organization, cellular protein catabolic process, protein localization to the membrane, the regulation of protein serine/threonine kinase activity, developmental growth, positive regulation of cell death, head development, and the negative regulation of cell proliferation.

We further overlapped the 2737 methylation marker genes with the genes from the Human Cancer Metastasis Database (HCMDB) [37]. The overlap analysis resulted in 383 genes. The top enriched GO and pathway terms are for 383 metastatic genes, including pathways in cancer, the regulation of cell adhesion, the negative regulation of cell proliferation, apoptotic signaling pathway, blood vessel morphogenesis, the positive regulation of cell death, the positive regulation of cell motility, the regulation of growth, epithelial cell proliferation, the regulation of cellular response to stress, the negative regulation of cell differentiation, response to wounding, tissue morphogenesis, heart development, transmembrane receptor protein tyrosine kinase signaling pathway, taxis, response to growth factor, the regulation of cytokine production, and the VEGFA-VEGFR2 signaling pathway.

Next, we performed protein–protein interaction (PPI) enrichment analysis using the molecular complex detection (MCODE) [39] module of Metascape for the 383 metastatic biomarker genes. The PPI enrichment analysis resulted in a network (Figure S5A) characterized by the presence of 11 PPI modules, including 147 genes of which 10 genes (*ABL1*, *SHC1, CDKN1A*, *LIMK1*, *SFRP1*, FASLG, *EDNRB*, *TNFRSF10B*, *OCLN,* and *SEMA5A*) were defined as seed genes (Figure S5B, Supplementary Table S6). Pathways in cancer, the regulation of cell adhesion, and the apoptotic signaling pathway are the top 3 enriched terms.

Furthermore, we overlapped 2737 methylation biomarker genes as well as 383 metastatic-specific genes from our study with the pathology atlas component of HPA [38]. We used the criteria protein expression score based on immunohistochemical analysis as a strong indicator to define the overlap. We detected 49 genes out of 2737 biomarker genes from our study that overlapped with HPA genes, while 16 genes out of the 383 metastatic-specific genes overlapped with the HPA genes (Table S7).

### 3.6. Comparison of Prediction Biomarkers with Similar Studies

We analyzed the overlap of 2978 methylation biomarkers used for cancer type prediction from our study with the published studies predicting tumor origin based on CpG biomarkers (Figure S5). A total of 20,451 CpGs were used for predicting the origin tissues based on tumor cell lines by Zhang et al. [40] using the minimum redundancy maximum relevance (mRMR) and Monte Carlo feature selection (MCFS) approaches. Next, we extracted 5709 CpGs from Tang et al.’s study [24], which discriminates tumor origin using a machine learning approach. In addition, we compared our methylation biomarkers with 10,360 CpGs from the work of Zheng et al. [5]. According to our Venn analysis, we found 165, 34, and 1164 CpGs from Zhang et al., Tang et al., and Zheng et al., respectively, overlapping with those from our studies (Table S8). Many prediction biomarkers used in our study overlapped with those from Zheng et al. This is perhaps due to the more considerable overlap with the data sets used as well as the more significant number of CpG biomarkers used for cancer prediction by Zheng et al. Overall, from our comparison analysis, we found common CpG biomarkers, from the studies compared for predicting tissue origin, irrespective of the methods employed. Thus, the results suggest the overlapping biomarkers can be an important candidate for cancer type prediction. However, these biomarker candidates require further clinical validation.

## 4. Discussion

The current study aimed to classify primary and metastatic cancer types using DNA methylation patterns by considering their tissue of origin. We selected methylation biomarkers as features for machine learning classification using differential methylation analysis, resulting in a linear model fit. The ability of methylation biomarkers to cluster the samples according to tissue types was visualized using a t-SNE plot.

Most related studies identifying methylation markers focus on one or only a few cancer types. We attempted to classify three classes (normal tissues and primary and metastatic cancers) for every tissue type using a multi-label machine learning classifier. We explored decision tree-based classifiers, including XGBoost, RF, probability-based classifier NB, and SVM in this study. Since each machine learning method has its pros and cons, it is important to compare each approach with the others in terms of their performance metrics. We observed that XG BOOST and SVM had the highest accuracy. Some of the samples in our data set had a very low number of samples per class. For example, there were only five samples from metastatic thyroid cancer and only eight normal samples originating from the rectum. Therefore, we performed oversampling using SMOTE, which resulted in a balanced sample set for the classification, improving the overall prediction accuracy (99%). Moreover, the sensitivity and specificity reached over 98% for XGBoost and RF. However, RF outperformed all the classifiers, with overall performance metrics of 99%. We also noted that the test set’s sensitivity and specificity for all the classes tested were higher than 90%.

Furthermore, our classifier showed an excellent performance in predicting primary cancer types, normal samples, and metastatic cancer types. In addition, analysis with LIME enabled us to demonstrate the reasoning behind the classifier’s decision for every instance. The LIME results depicted *BANK1* (cg00332153) as one of the top features for classifying primary breast cancer (Figure 3). In a previous study by Salhia et al. [41], it was suggested that BANK1 gene methylation is related to brain metastasis. Similarly, *HOXD4* (cg01152019) has been shown as the top prediction biomarker in metastatic prostate adenocarcinoma by our study. According to the published studies, homeobox (HOX) transcription factors are related to cancer progression [42] as well as cancer predisposition [43].

The GO and pathway analyses revealed that the 2978 CpG sites with the most variable features chosen for the classifier training were significantly enriched in the normal tissue development, differentiation, and cancer-specific processes. This suggests our study identified epigenetic markers to determine both normal tissue classification and classification of primary and metastatic tumor types. Moreover, our biomarkers had a suitable corroboration with the genes presented in the pathology atlas component of the HPA database, containing experimentally validated IHC results.

Some of the potential drawbacks of the study must be highlighted. Firstly, we do not have sufficient metastatic and normal samples to perform a robust classification, though the SMOTE approach provided balanced data sets, resulting in higher accuracy. Secondly, as acknowledged by Zheng et al. [5], the number of samples for the prediction of cancers of unknown primary (CUP) origin is very low in both the TCGA and GEO. Therefore, we could not use our classifier for the prediction of CUP. Thirdly, due to the lack of cell-free DNA (cfDNA), we could not extend the application of our classifier to cfDNA methylome samples. We also acknowledge that LIME explanations are not robust. In other words, even minor changes in the input vector of features may affect the LIME explanations dramatically in terms of signs of feature importance and the corresponding weights assigned to each of those features [44]. Although methylation-based biomarkers are a rapidly emerging approach for tumor detection, histology and IHC are still the most widely used approaches. Despite the fact that conventional histologic evaluation is still needed for identifying tumor origin, we believe that our results could be a suitable additional tool for clinicians to decide better treatment options for patients when a pathology-based approach fails.

## 5. Conclusions

This study aimed to classify cancer subtypes using machine learning based on DNA methylation data from the TCGA and GEO by considering the cancer tissue of origin. As a result, the RF classifier showed the highest performance for classifying different cancer types. In addition to the interpretation of the classifier using LIME, we demonstrated the biological importance of our analysis by performing GO and KEGG pathway analyses. We believe that the proposed model will be a helpful tool for cancer diagnosis, prognosis, and patient stratification. The combination of AI-based approaches and methylome profiling data will be valuable in the prediction of cancer types. Moreover, the increased accuracy offered by this AI-based method indicates its potential for cost-effectiveness and the feasibility of its use.

## Figures and Tables

**Table 1:** Performance of the RF multi-label classification model after performing SMOTE for the prediction of every tissue/cancer type. Tissue/cancer type prediction, tissue origin, and labels are included in the table, along with the performance metrics. BLCA: bladder urothelial carcinoma; BRCA: breast invasive carcinoma; CESC: cervical squamous cell carcinoma and endocervical adenocarcinoma; CHOL: cholangiocarcinoma; COAD: colon adenocarcinoma; ESCA: esophageal carcinoma; GBM: glioblastoma multiforme; HNSC: head and neck squamous cell carcinoma; KIRC: kidney renal clear cell carcinoma; KIRP: kidney renal papillary cell carcinoma; LIHC: liver hepatocellular carcinoma; LUAD: lung adenocarcinoma; LUSC: lung squamous cell carcinoma; OV: ovarian cancer; PAAD: pancreatic adenocarcinoma; PCPG: pheochromocytoma and paraganglioma; PRAD: prostate adenocarcinoma; READ: rectum adenocarcinoma; SARC: sarcoma; SKCM: skin cutaneous melanoma; STAD: stomach adenocarcinoma; THCA: thyroid carcinoma; THYM: thymoma; UCEC: uterine corpus endometrial carcinoma.

Label	Tissue Origin	Cancer Type Prediction (Normal Tissue/Primary Cancer/Metastatic Cancer)	Precision	Recall	F1-Score	Accuracy
BLCA.Normal	Bladder	Normal	1.0	1.0	1.0	1.0
BLCA.Primary	Bladder	Primary	0.994	0.956	0.974	1.0
BRCA.Metastatic	Breast	Metastatic	0.973	1.0	0.986	1.0
BRCA.Normal	Breast	Normal	0.987	1.0	0.993	1.0
BRCA.Primary	Breast	Primary	0.976	0.932	0.954	0.926
CESC.Primary	Cervix	Primary	0.971	0.982	0.977	0.952
CHOL.Normal	Bile duct	Normal	1.0	1.0	1.0	1.0
CHOL.Primary	Bile duct	Primary	1.0	1.0	1.0	1.0
COAD.Metastatic	Colon and/or rectum	Metastatic	1.0	1.0	1.0	1.0
COAD.Normal	Colon and/or rectum	Normal	1.0	1.0	1.0	1.0
COAD.Primary	Colon and/or rectum	Primary	0.993	0.980	0.987	0.98
ESCA.Normal	Esophagus	Normal	1.0	1.0	1.0	1.0
ESCA.Primary	Esophagus	Primary	0.982	0.921	0.951	0.975
GBM.Normal	Brain	Normal	1.0	1.0	1.0	1.0
GBM.Primary	Brain	Primary	1.0	1.0	1.0	1.0
HNSC.Normal	Head and Neck	Normal	0.994	1.0	0.997	1.0
HNSC.Primary	Head and Neck	Primary	0.921	0.926	0.924	0.950
KIRC.Normal	Kidney	Normal	1.0	0.993	0.996	1.0
KIRC.Primary	Kidney	Primary	1.0	0.974	0.987	0.993
KIRP.Normal	Kidney	Normal	0.993	1.0	0.996	1.0
KIRP.Primary	Kidney	Primary	0.986	1.0	0.993	0.989
LIHC.Normal	Liver	Normal	1.0	1.0	1.0	1.0
LIHC.Primary	Liver	Primary	1.0	0.993	0.996	0.996
LUAD.Normal	Lung	Normal	1.0	1.0	1.0	1.0
LUAD.Primary	Lung	Primary	0.993	0.993	0.993	0.974
LUSC.Normal	Lung	Normal	0.993	1.0	0.996	1.0
LUSC.Primary	Lung	Primary	0.910	0.953	0.931	0.951
OV.Metastatic	Ovary	Metastatic	1.0	1.0	1.0	1.0
OV.Normal	Ovary	Normal	1.0	1.0	1.0	1.0
OV.Primary	Ovary	Primary	1.0	1.0	1.0	1.0
PAAD.Normal	Pancreas	Normal	1.0	1.0	1.0	1.0
PAAD.Primary	Pancreas	Primary	0.993	0.986	0.989	0.981
PCPG.Primary	Adrenal gland	Primary	1.0	1.0	1.0	1.0
PRAD.Metastatic	Prostate	Metastatic	1.0	1.0	1.0	1.0
PRAD.Normal	Prostate	Normal	0.980	1.0	0.990	0.982
PRAD.Primary	Prostate	Primary	1.0	0.980	0.989	0.988
READ.Normal	Rectum	Normal	1.0	1.0	1.0	1.0
READ.Primary	Rectum	Primary	0.992	1.0	0.996	1.0
SARC.Primary	Soft tissue	Primary	0.970	1.0	0.985	1.0
SKCM.Metastatic	Skin	Metastatic	0.986	0.980	0.983	0.974
SKCM.Primary	Skin	Primary	0.980	1.0	0.990	0.993
STAD.Primary	Stomach	Primary	0.944	0.964	0.954	0.939
THCA.Metastatic	Thyroid	Metastatic	1.0	1.0	1.0	1.0
THCA.Normal	Thyroid	Normal	1.0	1.0	1.0	1.0
THCA.Primary	Thyroid	Primary	1.0	1.0	1.0	0.993
THYM.Primary	Thymus	Primary	1.0	1.0	1.0	1.0
UCEC.Metastatic	Uterus	Metastatic	0.971	1.0	0.985	1.0
UCEC.Normal	Uterus	Normal	0.993	1.0	0.996	1.0
UCEC.Primary	Uterus	Primary	0.987	0.970	0.978	0.961
Average accuracy						0.981

## Data Availability

The data that support the findings of this study are openly available in the TCGA database accessible via the Xena Browser (https://xena.ucsc.edu/, last accessed 1 April 2021). Source code used in this study is publicly available in a Github repository (https://github.com/shakshi12/AI-Explainability---BioInformatics, accessed on 15 May 2021. A Jupyter Notebook to replicate all the machine learning experiments used in the study is also available in the Github repository.

## Supplementary Materials

The following are available online at https://www.mdpi.com/article/10.3390/cancers13153768/s1, Figure S1: Distributed stochastic neighbor embedding (t-SNE) plot for the smallest hybrid model based on information from 2978 CpG sites, Figure S2: Principal component analysis (PCA) plot based on 2978 CpG sites used as the prediction biomarkers, Figure S3: A receiver operating characteristic curve (ROC) for cancer type prediction after performing synthetic minority oversampling technique (SMOTE), Figure S4: Results of LIME for top 10 biomarkers using RF classifier illustrated for metastatic cancer types, Figure S5: PPI network for the metastatic methylation biomarkers, Figure S6: Venn diagram of 2978 CpG sites used as prediction biomarkers in comparison with similar studies predicting cancer origin based on DNA methylation patters, Table S1: Methylome samples used in this study for the classification, Table S2: Description of 2978 CpG sites used as prediction biomarkers, Table S3: Results of the five-fold machine learning classifier results before and after performing SMOTE, Table S4: Confusion matrix for cancer type prediction after performing synthetic minority oversampling technique (SMOTE), Table S5: Enriched terms using GO and KEGG analysis performed using Metascape, Table S6: PPI enrichment analysis using the molecular complex detection (MCODE) module of Metascape for the 383 metastatic biomarker genes, Table S7: Genes from HPA pathology database overlapping with prediction biomarkers and metastatic-specific biomarkers, Table S8: Overlapping CpGs consisting of 2978 methylation biomarkers used for the cancer type prediction from our study with similar studies.
