# Supplementary material for: Machine Learning Approaches to Classify Primary and Metastatic Cancers Using Tissue of Origin-Based DNA Methylation Profiles
Source: Cancers (Basel). 2021 Jul 27;13(15):3768. doi: 10.3390/cancers13153768 (PMC8345047; doi:10.3390/cancers13153768)
Supplement: Supplementary file 1 [file cancers-13-03768-s001.zip › cancers-1253923-supplementary final2/Supplementary Figures.pdf]

Supplementary Figures: Machine Learning Approaches to Classify Primary and Metastatic Cancers Using Tissue of Origin-Based DNA Methylation Profiles

Vijayachitra Modhukur, Shakshi Sharma, Mainak Mondal, Ankita Lawarde, Keiu Kask, Rajesh Sharma and Andres Salumets

Supplementary Figures

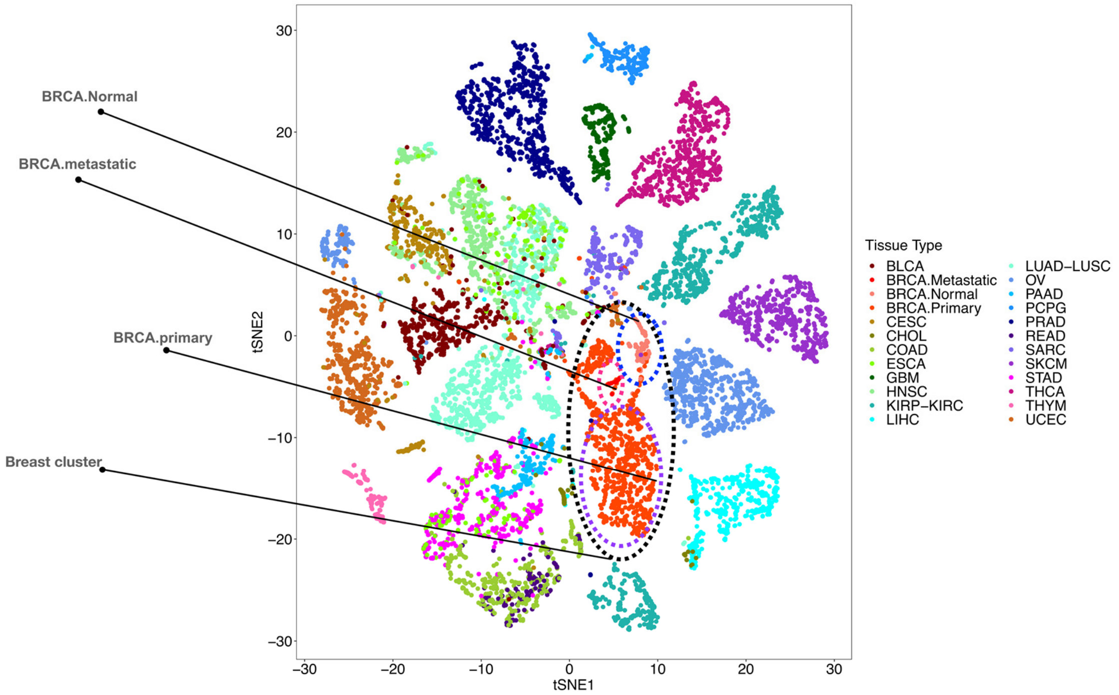

Figure S1. Distributed stochastic neighbor embedding (t-SNE) plot for the smallest hybrid model based on information from 2978 CpG sites. BLCA: Bladder Urothelial Carcinoma; BRCA: Breast Invasive Carcinoma; CESC: Cervical squamous cell carcinoma and endocervical adenocarcinoma; CHOL: Cholangiocarcinoma; COAD: Colon adenocarcinoma; ESCA: Esophageal carcinoma; GBM: Glioblastoma multiforme; HNSC: Head and Neck squamous cell carcinoma; KIRC: Kidney renal clear cell carcinoma; KIRP: Kidney renal papillary cell carcinoma; LIHC: Liver hepatocellular carcinoma; LUAD: Lung adenocarcinoma; LUSC: Lung squamous cell carcinoma; OV: Ovarian cancer; PAAD: Pancreatic adenocarcinoma; PCPG: Pheochromocytoma and Paraganglioma; PRAD: Prostate adenocarcinoma; READ: Rectum adenocarcinoma; SARC: Sarcoma; SKCM: Skin Cutaneous Melanoma; STAD: Stomach adenocarcinoma; THCA: Thyroid carcinoma; THYM: Thymoma; UCEC: Uterine Corpus Endometrial Carcinoma. We can see from the t-SNE plot that samples are clustered according to the tissue types. For example, ovarian samples were clustered together (shown in light green), liver samples were clustered together (shown in light pink) and esophagus samples were clustered together (shown in purple). Cluster formed from breast tissue is highlighted, where normal, primary and metastatic sample types are highlighted.

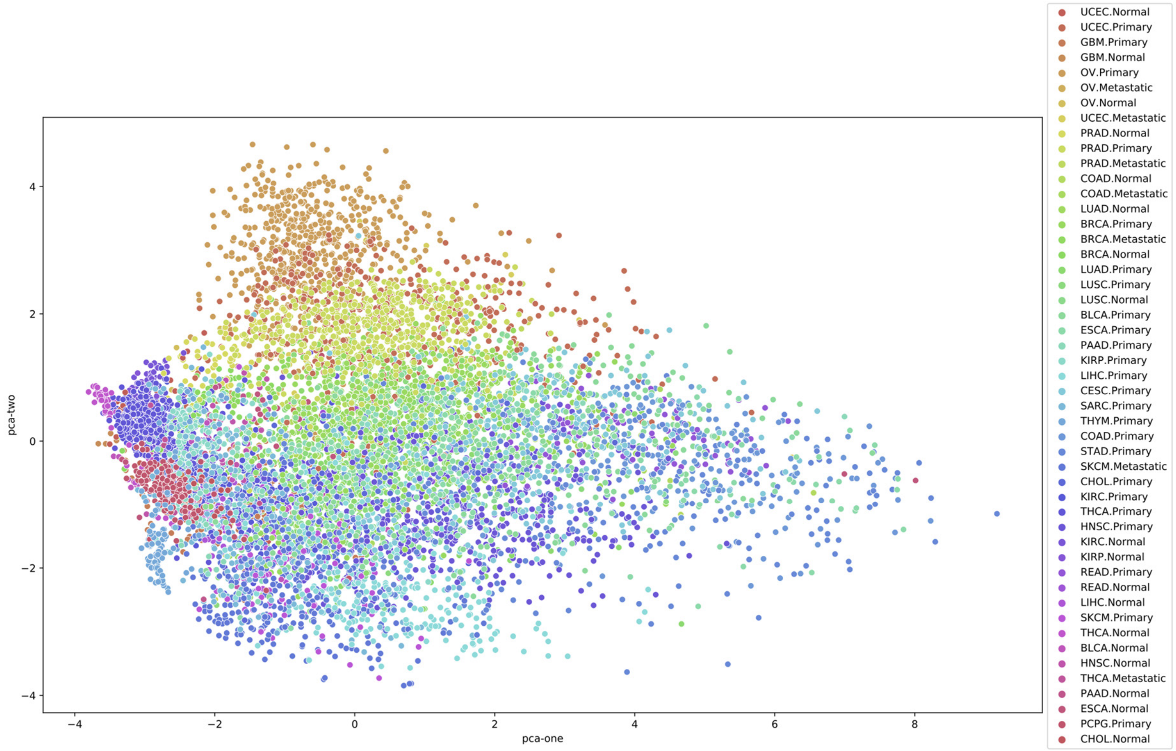

Figure S2. Principal Component Analysis (PCA) plot based on 2,978 CpG sites used as the prediction biomarkers. Every tissue type is denoted by unique color. BLCA: Bladder Urothelial Carcinoma; BRCA: Breast Invasive Carcinoma; CESC: Cervical squamous cell carcinoma and endocervical adenocarcinoma; CHOL: Cholangiocarcinoma; COAD: Colon adenocarcinoma; ESCA: Esophageal carcinoma; GBM: Glioblastoma multiforme; HNSC: Head and Neck squamous cell carcinoma; KIRC: Kidney renal clear cell carcinoma; KIRP: Kidney renal papillary cell carcinoma; LIHC: Liver hepatocellular carcinoma; LUAD: Lung adenocarcinoma; LUSC: Lung squamous cell carcinoma; OV: Ovarian cancer; PAAD: Pancreatic adenocarcinoma; PCPG: Pheochromocytoma and Paraganglioma; PRAD: Prostate adenocarcinoma; READ: Rectum adenocarcinoma; SARC: Sarcoma; SKCM: Skin Cutaneous Melanoma; STAD: Stomach adenocarcinoma; THCA: Thyroid carcinoma; THYM: Thymoma; UCEC: Uterine Corpus Endometrial Carcinoma. .

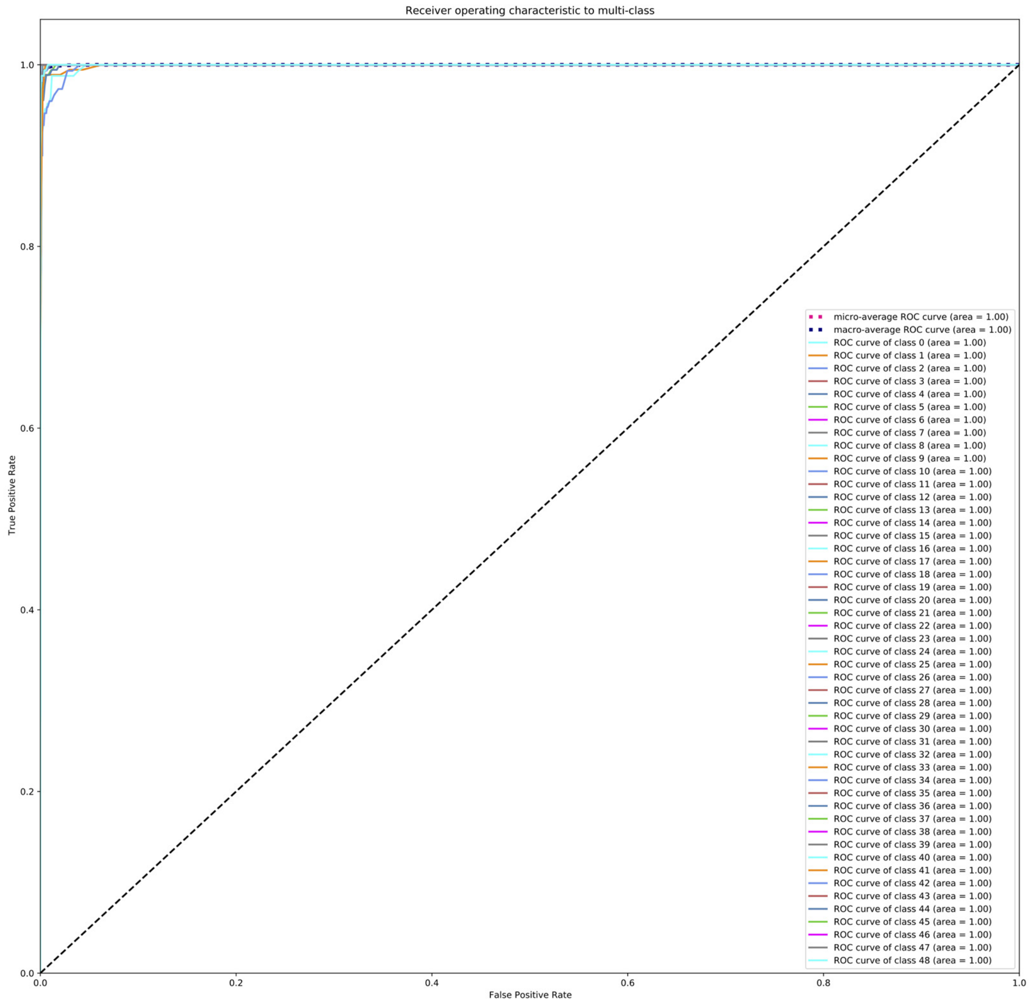

Figure S3. A receiver operating characteristic curve (ROC) for cancer type prediction after performing Synthetic Minority Oversampling Technique (SMOTE). Class 0=BLCA.Normal; Class 1=BLCA.Primary; Class 2=BRCA.Metastatic; Class 3=BRCA.Normal; Class 4=BRCA.Primary; Class 5=CESC.Primary; Class 6=CHOL.Normal; Class 7=CHOL.Primary; Class 8=COAD.Metastatic; Class 9=COAD.Normal; Class 10=COAD.Primary; Class 11=ESCA.Normal; Class 12=ESCA.Primary; Class 13=GBM.Normal; Class 14=GBM.Primary; Class 15=HNSC.Normal; Class 16=HNSC.Primary; Class 17=KIRC.Normal; Class 18=KIRC.Primary; Class 19=KIRP.Normal; Class 20=KIRP.Primary; Class 21=LIHC.Normal; Class 22=LIHC.Primary; Class 23=LUAD.Normal; Class 24=LUAD.Primary; Class 25=LUSC.Normal; Class 26=LUSC.Primary; Class 27=OV.Metastatic; Class 28=OV.Normal; Class 29=OV.Primary; Class 30=PAAD.Normal; Class 31=PAAD.Primary; Class 32=PCPG.Primary; Class 33=PRAD.Metastatic; Class 34=PRAD.Normal; Class 35=PRAD.Primary; Class 36=READ.Normal; Class 37=READ.Primary; Class 38=SARC.Primary; Class 39=SKCM.Metastatic; Class 40=SKCM.Primary; Class 41=STAD.Primary; Class 42=THCA.Metastatic; Class 43=THCA.Normal; Class 44=THCA.Primary; Class 45=THYM.Primary; Class 46=UCEC.Metastatic; Class 47=UCEC.Normal; Class 48=UCEC.Primary.

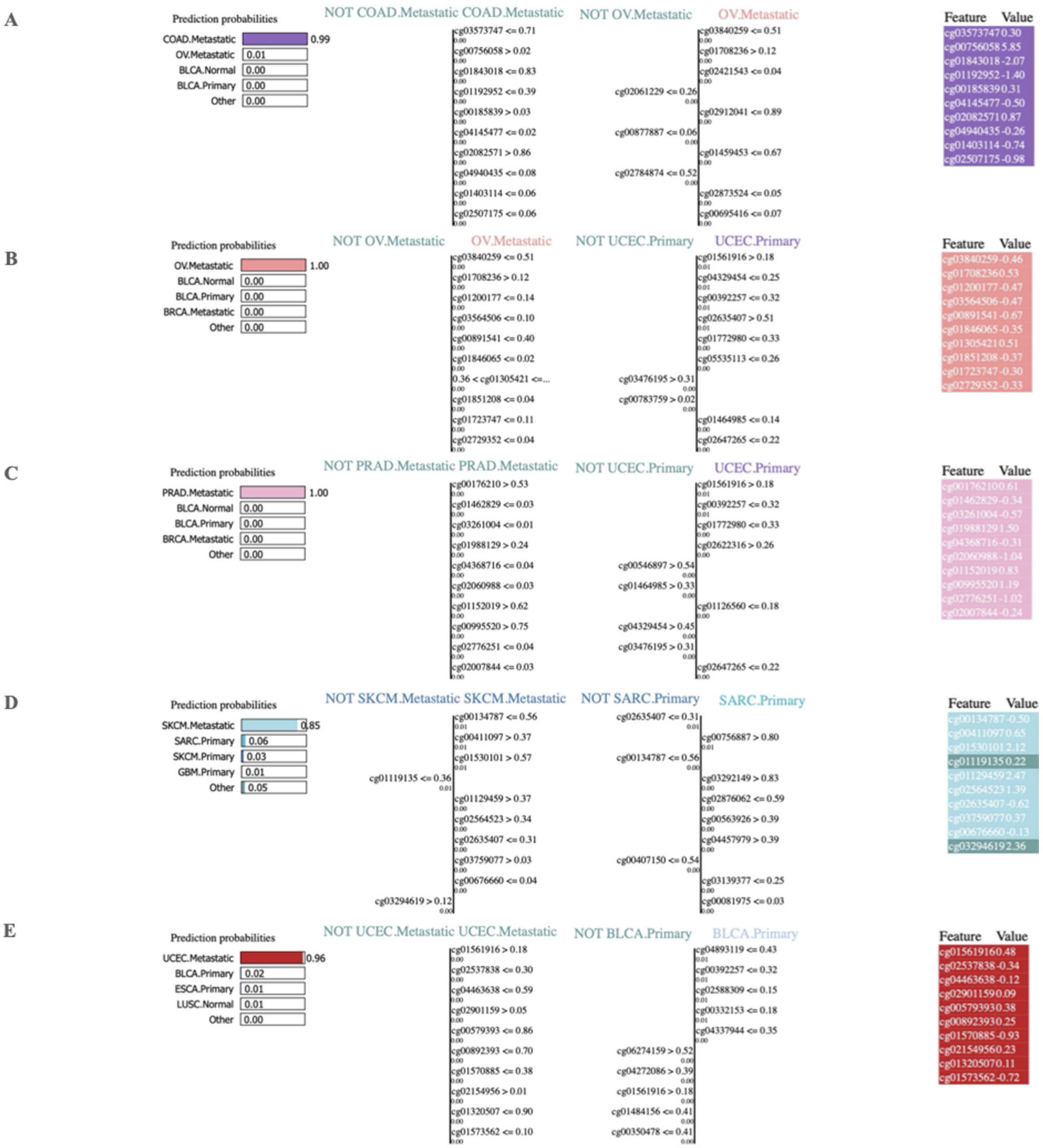

Figure S4. Results of LIME for top 10 biomarkers using RF classifier illustrated for metastatic cancer types. (A) Lime interpretations for metastatic colon adenocarcinoma. The violet color denotes the positive instance. The first column represents the prediction probabilities of negative and positive results achieved from the classifiers. The second column shows the features' contributions to the probability. The third column displays the original data values. (B) LIME interpretation for metastatic ovarian cancer. (C) LIME interpretation for metastatic Prostate adenocarcinoma. (D) LIME interpretation for metastatic skin cutaneous melanoma. (E) LIME interpretation for metastatic uterine corpus endometrial carcinoma. LIME: local interpretable model-agnostic explanations; RF: random forest.

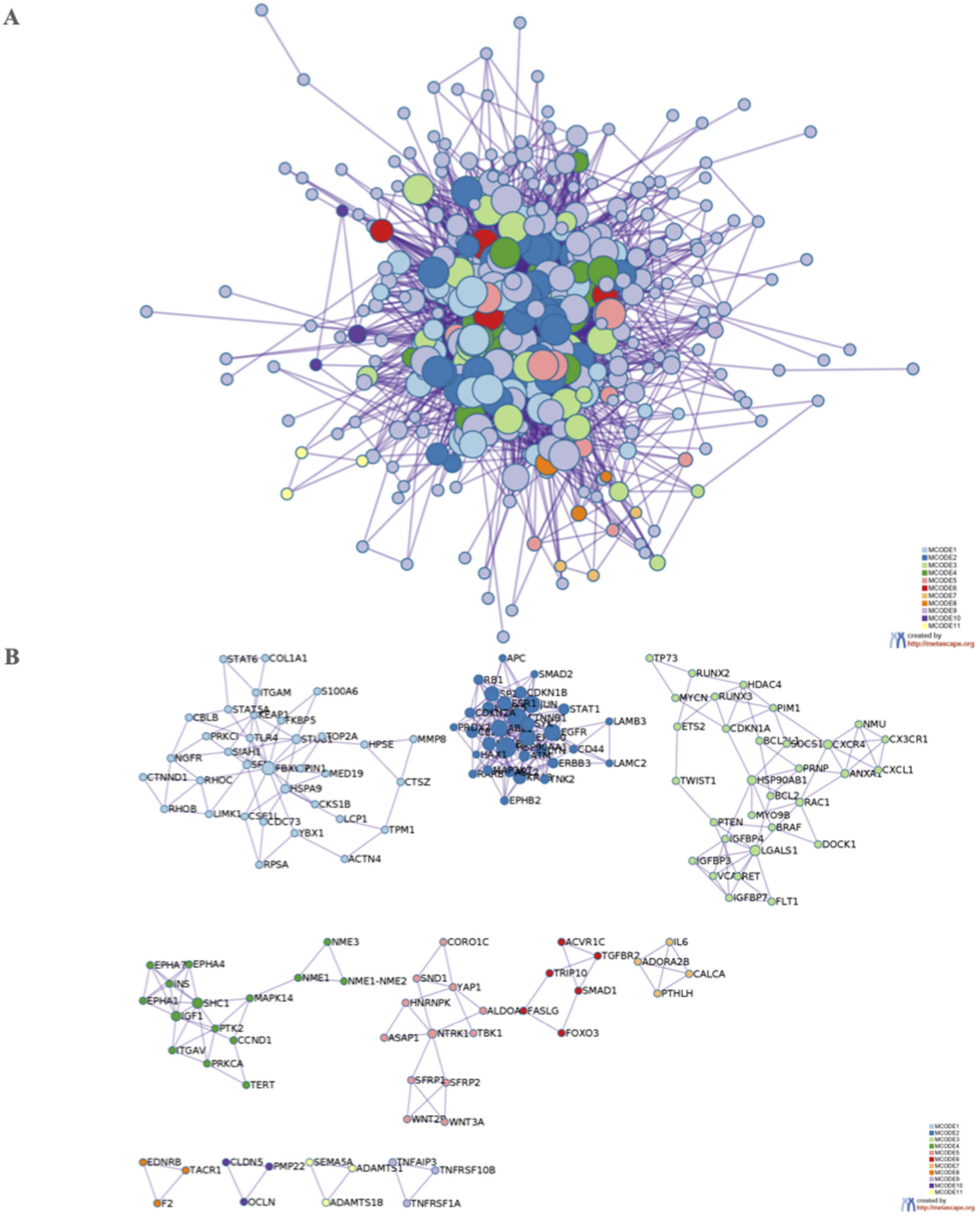

Figure S5. PPI network for the metastatic methylation biomarkers resulting from (**A**) the MCODE module of Metascape analysis, detected 11 modules represented by a unique color and the (**B**) corresponding MCODE gene components identified in the PPI network.

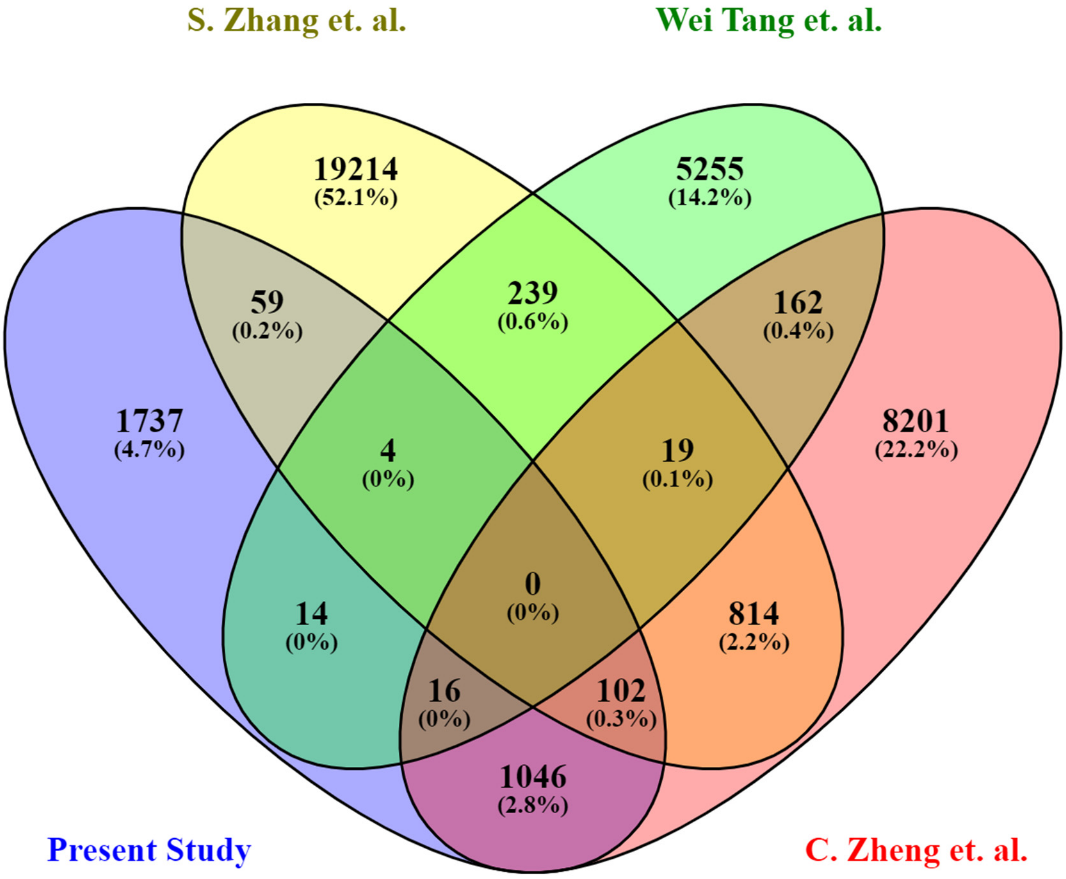

Figure S6. Venn diagram of 2,978 CpG sites used as prediction biomarkers in comparison with similar studies predicting cancer origin based on DNA methylation patters. The venn diagram was generated using the webtool 'venny' (http://bioinfogp.cnb.csic.es/tools/venny/).

.

Please View the Supplementary Tables at the Excel Files.

Table S1: Methylome samples used in this study for the classification, Table S2: Description of 2,978 CpG sites used as prediction biomarkers, Table S3: Results of the five-fold machine learning classifier results before and after performing SMOTE, Table S4: Confusion matrix for cancer type prediction after performing Synthetic Minority Oversampling Technique (SMOTE), Table S5: Enriched terms using GO and KEGG analysis performed using Metascape, Table S6: (PPI) enrichment analysis using the Molecular Complex Detection (MCODE) module of Metascape for the 383 metastatic biomarker genes, Table S7: Genes from HPA pathology database overlapping with prediction biomarkers and metastatic specific biomarkers, Table S8. Overlapping CpGs consisting of 2,978 methylation biomarkers used for the cancer type prediction from our study with similar studies.
